# Neuroprotective Effect of the Inhibitor Salubrinal after Cardiac Arrest in a Rodent Model

**DOI:** 10.1155/2020/7468738

**Published:** 2020-01-22

**Authors:** Jincheng Zhang, Yang Wang, Minjie Ju, Jieqiong Song, Yijuan Zheng, Shilong Lin, Duming Zhu, Lu Wen, Ming Zhong, Shuming Pan, Guangtian Yang

**Affiliations:** ^1^Department of Critical Care Medicine, Zhongshan Hospital, Fudan University, Shanghai 200032, China; ^2^Department of Emergency, Xinhua Hospital, Shanghai Jiao Tong University School of Medicine, Shanghai 200092, China; ^3^Department of Otolaryngology, Shanghai Jiao Tong University Affiliated Sixth People's Hospital, Shanghai Jiao Tong University School of Medicine, Shanghai, China; ^4^Department of Emergency, Tongji Hospital, Tongji Medical College, Huazhong University of Science and Technology, Wuhan 430030, China

## Abstract

Cardiac arrest (CA) yields poor neurological outcomes. Salubrinal (Sal), an endoplasmic reticulum (ER) stress inhibitor, has been shown to have neuroprotective effects in both in vivo and in vitro brain injury models. This study investigated the neuroprotective mechanisms of Sal in postresuscitation brain damage in a rodent model of CA. In the present study, rats were subjected to 6 min of CA and then successfully resuscitated. Either Sal (1 mg/kg) or vehicle (DMSO) was injected blindly 30 min before the induction of CA. Neurological status was assessed 24 h after CA, and the cortex was collected for analysis. As a result, we observed that, compared with the vehicle-treated animals, the rats pretreated with Sal exhibited markedly improved neurological performance and cortical mitochondrial morphology 24 h after CA. Moreover, Sal pretreatment was associated with the following: (1) upregulation of superoxide dismutase activity and a reduction in maleic dialdehyde content; (2) preserved mitochondrial membrane potential; (3) amelioration of the abnormal distribution of cytochrome C; and (4) an increased Bcl-2/Bax ratio, decreased cleaved caspase 3 upregulation, and enhanced HIF-1*α* expression. Our findings suggested that Sal treatment improved neurological dysfunction 24 h after CPR (cardiopulmonary resuscitation), possibly through mitochondrial preservation and stabilizing the structure of HIF-1*α*.

## 1. Introduction

Despite the obvious advancement in the field of cardiac arrest (CA), global brain damage that involves a complex cascade of events remains a leading cause of mortality in patients after CPR [1]. To date, mild therapeutic hypothermia is considered the only effective therapy for cerebral ischemia reperfusion injury, but it has not yet been shown to lead to high levels of neurological functional recovery [2–5]. Therefore, developing new treatments to improve the survival of patients with CA remains a major goal.

Recent studies have demonstrated that endoplasmic reticulum (ER) stress is closely connected with the process of ischemia/reperfusion (I/R) injury [6]. The endoplasmic reticulum is a multifunctional organelle that plays important roles in multiple biological processes, including lipid biosynthesis, protein folding, and Ca^2+^ storage [7]. The accumulation of unfolded or misfolded proteins in the ER lumen disrupts ER homeostasis and activates ER stress [8]. Moderate ER stress can increase the chance of cell survival, but prolonged and severe ER stress can trigger apoptosis and lead to cell death [9].

We and other investigators have previously shown that ER stress and its related apoptotic signaling pathways are activated in brain injury after CPR [10–12]. Salubrinal (Sal) is a specific ER stress inhibitor [13]. In our previous study [10], we administered Sal before CA and demonstrated that Sal can markedly moderate cell apoptosis and neurological dysfunction after CPR. Moreover, we observed that mitochondrial damage was reduced. However, the mechanisms remain unclear. Hence, we used a 6 min CA rat model to study the effects of Sal on brain mitochondria and the possible underlying mechanisms.

## 2. Materials and Methods

Male Sprague-Dawley rats were raised under standard laboratory conditions with free access to water and food. The experimental protocol was approved by the Institutional Animal Care and Use Committee of Xinhua Hospital Affiliated Shanghai Jiao Tong University School of Medicine (approval no. XFEC-F-2016-090) and Huazhong University of Science and Technology (no. S281).

The method of CA induction and resuscitation in this experiment was previously well described by Lin et al. [14] and has been used extensively in our lab [10, 15–17]. Briefly, two disposable acupuncture needles were placed between the fourth rib and the right sternum border at the edge of the left sternum. A stimulating electrode was connected and delivers a 2 mA current of 60 Hz. Circulatory arrest was defined as a mean arterial blood pressure (MAP) ≤ 20 mmHg, pulseless electrical activity, and asystole or ventricular fibrillation on electrocardiogram when electrical stimulation stopped. The current flow lasted for 3 min to prevent spontaneous defibrillation. After 6 min of CA, advanced life support, specifically mechanical ventilation (100% FiO_2_, 0.65 mL/100 g and 100 breathes/min), chest compression (200 times/min), and adrenaline (2 *μ*g/100 g, iv), was provided. The return of spontaneous circulation (ROSC) was confirmed by an organized rhythm with an MAP ≥ 60 mmHg for at least 10 min. After ROSC, hemodynamic monitoring and mechanical ventilation were continued using 21% oxygen for 2 h. After recovery from anesthesia, the animals were returned to their cages and closely observed.

### 2.1. Administration of Salubrinal

Salubrinal (Sal), purchased from Santa Cruz Biotechnology (Calif), was dissolved in dimethyl sulfoxide (DMSO) and administered at a dose of 1 mg/kg (intraperitoneal) 30 min prior to the induction of CA [10]. For the vehicle-treated group, an equal volume of DMSO was administered 30 min before the induction of CA. In this study, a dose of 1 mg/kg of Sal was selected because this dose of Sal exerts a potent neuroprotective effect according to previous studies [6, 18].

### 2.2. Experimental Design

A 24 h study was designed to investigate the effects of Sal on neurological function in rats after CPR. Before the induction of CA, animals were assigned to the Sal or vehicle group by a random sequence generator. Registration and randomization were performed until 15 rats per group survived for 24 h. Neurological performance was assessed 24 h after CPR. The rats were then euthanized, and the brain tissues were removed for analysis by terminal deoxynucleotidyl transferase dUTP nick-end labeling (TUNEL) and transmission electron microscopy (TEM); oxidative stress, mitochondrial membrane potential (MMP), and cytochrome C were detected.

### 2.3. Neurological Deficit Scores

Neurological deficit scores (NDSs) were assessed 24 h after CPR. NDSs were determined as described previously [19]. Briefly, the NDS scale consisted of 5 components: general behavioral function (40 points), cranial nerve function (20 points), motor function (10 points), sensory function (10 points), and coordination (20 points). The scoring system ranged from 0 (normal) to 100 (brain death). NDS was determined independently by two investigators blinded to treatment.

### 2.4. Neuron-Specific Enolase (NSE) and S100*β* in the Serum

Blood samples were collected from the femoral vein 24 h after CPR. Serum NSE and S100*β* levels were quantified with ELISA kits (Elabscience Biotechnology, China) according to the manufacturer's instructions.

### 2.5. TUNEL Staining

Terminal deoxynucleotidyl transferase dUTP nick-end labeling staining was performed in strict accordance with the manufacturer's instructions (Roche, USA). The investigators were blinded to the experimental groups. Cells stained brown were TUNEL-positive cells. For each specimen, the total number of cells with positive nuclear staining from 5 microscopic fields (×400) of the frontal cortex was counted. The data are expressed as the mean number of TUNEL-positive cells/the total number of cells per microscopic field of view.

### 2.6. Ultrastructural Analysis

Briefly, fresh brain tissues (frontal cortex, 1 mm^3^) were fixed in cold 2% glutaraldehyde (0.1 mol/L phosphate buffer, pH 7.4) and fixed in 1% buffered osmium tetroxide. The tissues were dehydrated with gradient ethanol solutions and embedded in epoxy resin. They were cut into ultrathin sections (80 nm) and stained with uranyl acetate and lead citrate. The micrographs were viewed under a TEM (Hitachi Ht7700, Japan). Pathology data were evaluated by independent investigators blinded to the experimental groups. All images are captured at ×5000 magnification.

### 2.7. Isolation of Mitochondria from the Frontal Cortex

Cortical mitochondria were isolated using a tissue mitochondria isolation kit (Beyotime Biotechnology, China). The assay was conducted according to the manufacturer's protocol. Mitochondrial preservation solution was added to separated mitochondria and gently mixed until the mitochondria were suspended. The collected supernatant was centrifuged at 12000 × g to detect cytochrome C.

### 2.8. Determination of the MMP

A mitochondrial membrane potential assay kit (Beyotime Biotechnology, China) with JC-1 was used. According to the manual, the fluorescence intensity of J-aggregates at 590 nm was measured using a fluorescence plate reader. The average fluorescence of 5 replicate wells minus that of the control wells was calculated.

### 2.9. Cytochrome C in the Cytosol

Cytochrome C was measured using an ELISA kit (Elabscience Biotechnology, China). The OD was measured using a microplate reader at a wavelength of 450 nm according to the manufacturer's protocol.

### 2.10. Oxidative Stress

Brain tissues (cortex) were collected 24 h after ROSC and then placed in ice-cold RIPA lysis buffer (Beyotime, Shanghai, China). The samples were then homogenized, followed by centrifugation at 11000 × g for 10 min. The supernatants were collected for malonaldehyde (MDA) and superoxide dismutase (SOD) measurements. A total SOD assay kit (Nanjing, Jiancheng Biochemical, China) and an MDA assay kit (Nanjing, Jiancheng Biochemical, China) were used to measure MDA and SOD. The assays were conducted according to the manufacturer's protocols.

### 2.11. Western Blotting

After 24 h of ROSC, the rats were euthanized and perfused. The brain tissues were removed, and frontal cortex tissues were immediately collected, frozen in liquid nitrogen, and stored at -80°C before use. Frozen cortex samples (50 mg) were homogenized in radioimmunoprecipitation assay lysis buffer (Beyotime, Shanghai, China), and the proteins were quantified using BCA assay reagents. A total of 30 *μ*g of protein from each sample was separated by 10% sodium dodecyl sulfate polyacrylamide gel electrophoresis and transferred to polyvinylidene fluoride membranes (Millipore, Billerica, Mass). The membranes were blocked with 5% nonfat milk for 2 h and incubated with primary antibodies at 4°C overnight. The following primary antibodies were used: a polyclonal rabbit anti-Bcl-2 antibody (1 : 1000; Proteintech Group, Chicago), a polyclonal rabbit anti-HIF-1*α* antibody (1 : 1000; Affinity, Cincinnati), a polyclonal rabbit anti-Bax antibody (1 : 1000; Proteintech Group, Chicago), a polyclonal rabbit anti-cleaved caspase 3 antibody (1 : 1000; Proteintech Group, Chicago), and a polyclonal rabbit anti-GAPDH antibody (1 : 2000; Proteintech Group, Chicago). After washing with TBS-T, the membranes were incubated in a suitable secondary antibody solution, and the blots were visualized and scanned. The immunoreactive bands were then analyzed using Image-Pro Plus (version 6.0). The quantitative protein data were normalized to GAPDH.

### 2.12. Statistical Analysis

Statistical analyses were performed using the SPSS statistics 23.0 program. The results are shown as the mean ± standard deviation (SD). Nonnormally distributed variables (neurological deficit scores) were analyzed by the Mann-Whitney *U* test. Normally distributed data were analyzed by one-way ANOVA and Student's *t*-test. *P* < 0.05 was considered significant.

## 3. Results

In the present study, CA was induced successfully in 50 rats, among which 2 rats in the vehicle group and 3 rats in the Sal group died after ROSC before reaching 24 h of survival.

The baseline characteristics of all animals in terms of hemodynamic, temperature, and respiratory parameters as well as lactate and glucose levels did not differ between the groups. However, CA and CPR resulted in marked biochemical and physiological disturbances in all groups. However, these changes gradually improved from CPR to mechanic ventilation weaning, and no difference was observed between the vehicle and Sal groups (Table 1).

### 3.1. Sal Improved Neurological Outcomes 24 h after CPR

**Figure 1:**
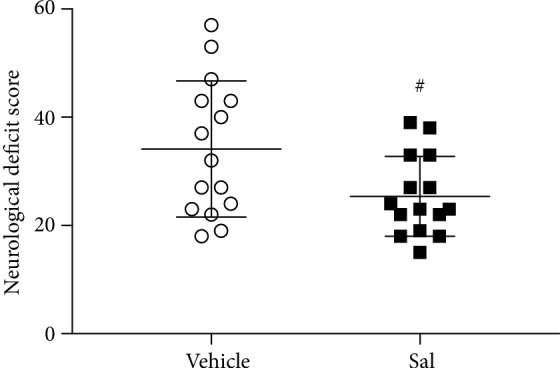
Neurological deficit scores of the vehicle and Sal groups 24 h after CA. Sal improved neurological outcomes 24 h after CA. ^#^*P* < 0.05 vs. the vehicle group.

As shown in Figure 1, there was an improvement in the NDS of the Sal group compared to that of the vehicle group after CA (25.4 ± 7.4 vs. 34.1 ± 12.6; *P* < 0.05). The NDS of each animal from all groups was zero prior to the induction of CA.

### 3.2. Sal Decreased the Upregulation of Serum NSE and S100*β* after CPR

**Figure 2:**
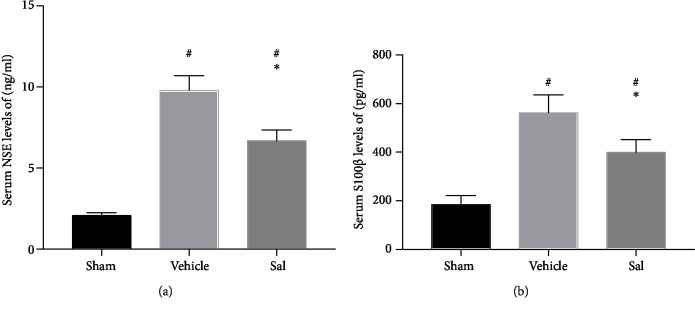
Effect of Sal on serum (a) NSE and (b) S100*β* levels 24 h after CA. Animals treated with Sal showed a lower level of NSE and S100*β* after CA. ^#^*P* < 0.01 vs. sham and ^∗^*P* < 0.01 vs. the vehicle group.

As presented in Figure 2, serum NSE and S100*β* levels were elevated significantly after CPR (NSE: sham: 2.06 ± 0.19 ng/mL, vehicle group: 9.76 ± 0.92 ng/mL, *P* < 0.01; S100*β*: sham: 184.05 ± 37.33 pg/mL, vehicle group: 561.68 ± 74.66 pg/mL, *P* < 0.01). However, this increase was significantly ameliorated by Sal (NSE: vehicle group: 9.76 ± 0.92 ng/mL, Sal group: 6.68 ± 0.67 ng/mL, *P* < 0.01; S100*β*: vehicle group: 561.68 ± 74.66 pg/mL, Sal group: 398.66 ± 52.94 pg/mL, *P* < 0.01).

### 3.3. Sal Reduced the Apoptosis of Cortical Cells after CPR

**Figure 3:**
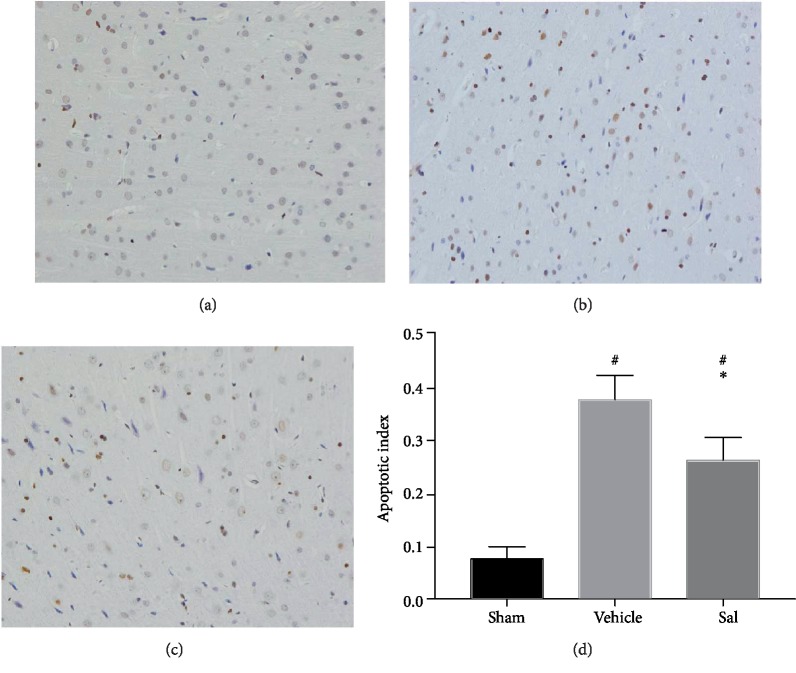
Effect of Sal on brain cell apoptosis in the cortex 24 h after CA. Representative images of TUNEL staining in the cortex of the (a) sham, (b) vehicle, and (c) Sal groups are shown. (d) The percentage of TUNEL-positive cells was significantly lower in the Sal group than in the vehicle group. The results are expressed as the mean ± SD. ^#^*P* < 0.05 vs. sham and ^∗^*P* < 0.05 vs. the vehicle group.

The percentage of TUNEL-positive cells was markedly lower in the Sal group than in the vehicle group 24 h after CA (vehicle: 0.37 ± 0.04, Sal: 0.26 ± 0.04, *P* < 0.05) (Figure 3).

### 3.4. Sal Mitigated the Damage to Cortical Mitochondria 24 h after CPR

**Figure 4:**
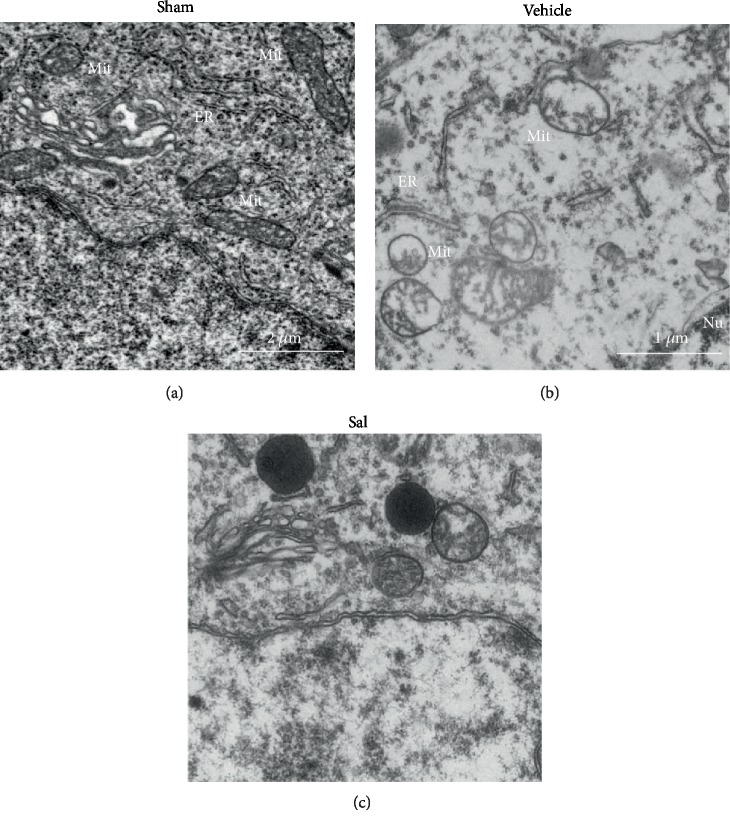
Ultrastructural changes in cortex mitochondria in the (a) sham, (b) vehicle, and (c) Sal groups 24 h after CPR. Sal mitigated the damage observed in cortical Mit 24 h after CPR. Mit: mitochondria; Nu: nucleus.

Typical electron micrographs of mitochondria in the frontal cortex of each of the 3 groups are shown in Figure 4. In the sham group, we observed little, if any, swelling and no membrane disruption (Figure 4(a)). CPR caused significant ultrastructural changes, including the loss of internal membrane contacts, disruption of mitochondrial cristae, and swelling of the mitochondrial matrix (Figure 4(b)). However, CA-induced mitochondrial changes were significantly improved upon treatment with Sal (Figure 4(c)).

### 3.5. Sal Decreased the Level of Oxidative Stress 24 h after CPR

**Figure 5:**
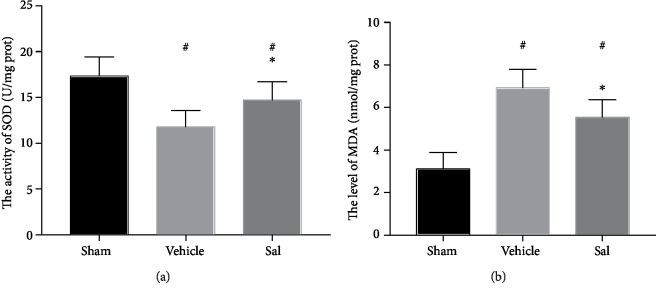
Effect of Sal on cortical (a) SOD activity and (b) MDA levels 24 h after CPR. Rats pretreated with Sal showed higher levels of SOD activity and lower levels of MDA. ^#^*P* < 0.01 vs. sham and ^∗^*P* < 0.01 vs. the vehicle group.

SOD activity after CA was markedly inhibited (sham: 17.34 ± 2.09 U/mg prot, vehicle group: 11.77 ± 1.80 U/mg prot, *P* < 0.01; sham: 17.34 ± 2.09 U/mg prot, Sal group: 14.70 ± 2.02 U/mg prot, *P* < 0.01), but SOD activity in the Sal group was increased compared to that in the vehicle group (vehicle group: 11.77 ± 1.80 U/mg prot, Sal group: 14.70 ± 2.02 U/mg prot, *P* < 0.01) (Figure 5(a)). Meanwhile, the MDA level after ROSC was higher than that in the sham group (sham: 3.11 ± 0.78 nmol/mg prot, vehicle group: 6.93 ± 0.86 nmol/mg prot, *P* < 0.01; sham: 3.11 ± 0.78 nmol/mg prot, Sal group: 5.54 ± 0.83 nmol/mg prot, *P* < 0.01). However, the MDA level in the vehicle group was higher than that in the Sal group (vehicle group: 6.93 ± 0.86 nmol/mg prot, Sal group: 5.54 ± 0.83 nmol/mg prot, *P* < 0.01) (Figure 5(b)).

### 3.6. Sal Inhibited the Excessive Hyperpolarization of the MMP 24 h after CPR

**Figure 6:**
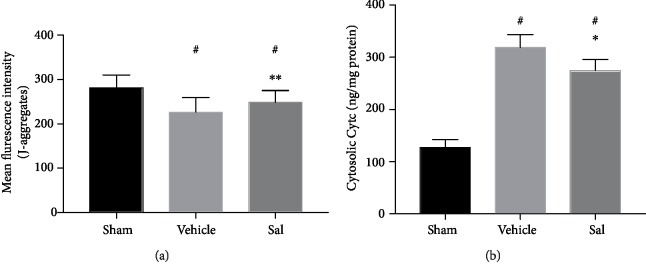
The mitochondrial membrane potential and cytochrome C leakage 24 h after CPR. Sal inhibited the excessive hyperpolarization of the MMP and mitigated the leakage of cytochrome C. ^#^*P* < 0.01 vs. sham, ^∗^*P* < 0.01 vs. the vehicle group, and ^∗∗^*P* < 0.05 vs. the vehicle group.

After CA and CPR, the MMP showed significant hyperpolarization (sham: 281.13 ± 28.94, vehicle group: 226.33 ± 33.16, *P* < 0.01). However, Sal administration before CA inhibited the excessive hyperpolarization of the MMP (vehicle group: 226.33 ± 33.16, Sal group: 251.67 ± 22.81, *P* < 0.05) (Figure 6(a)).

### 3.7. Sal Restrained the Leakage of Cytochrome C 24 h after CPR

After CA and CPR, the leakage of cytochrome C increased significantly (sham: 126.97 ± 15.44 ng/mg prot, vehicle group: 318.26 ± 25.22 ng/mg prot, *P* < 0.01). However, treatment with Sal before CA restrained the leakage of cytochrome C (vehicle group: 318.26 ± 25.22 ng/mg prot, Sal group: 274.30 ± 21.21 ng/mg prot, *P* < 0.01) (Figure 6(b)).

### 3.8. Sal Restored the Bcl-2/Bax Ratio, Increased HIF-1*α* Expression, and Decreased the Upregulation of Cleaved Caspase 3 in the Cortex 24 h after CPR

**Figure 7:**
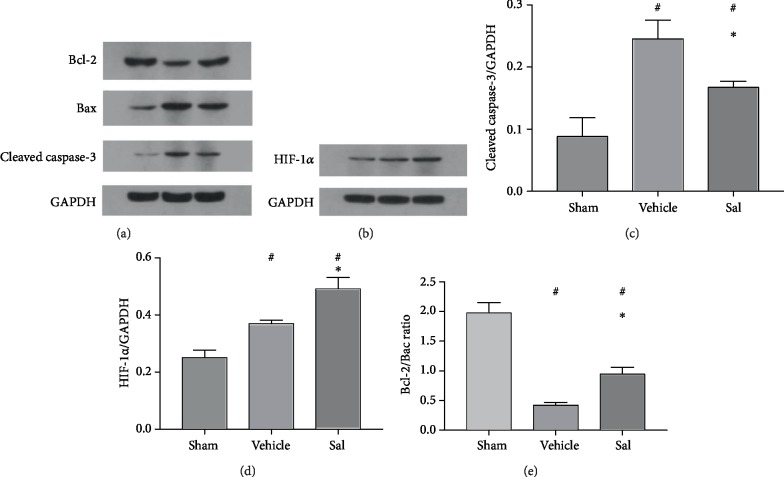
Expression of Bcl-2, Bax, cleaved caspase 3, and HIF-1*α* in the cortex at 24 h after CPR. (a, b) Representative western blots of Bcl-2, Bax, cleaved caspase 3, and HIF-1*α* are shown. Sal prevented (c) caspase 3 cleavage and increased the (d) expression of HIF-1*α* and the (e) Bcl-2/Bax protein ratio. ^#^*P* < 0.01 vs. sham and ^∗^*P* < 0.01 vs. the vehicle group.

After CPR, a decrease in the Bcl-2/Bax ratio and an increase in cleaved caspase 3 were observed in the rats (Figure 7(a)). However, Sal pretreatment restored the Bcl-2/Bax ratio (vehicle group: 0.42 ± 0.05, Sal group: 0.94 ± 0.12, *P* < 0.01) and decreased the upregulation of cleaved caspase 3 (vehicle group: 0.25 ± 0.03, Sal group: 0.17 ± 0.01, *P* < 0.01) in the cortex 24 h after CA. The expression of HIF-1*α* in the cortex increased after ROSC (sham group: 0.25 ± 0.03, vehicle group: 0.37 ± 0.01, *P* < 0.01). This increase was enhanced by Sal treatment (vehicle group: 0.37 ± 0.01, Sal group: 0.49 ± 0.04, *P* < 0.01) (Figure 7(d)).

## 4. Discussion

In our previous study, we demonstrated that ER stress is significantly activated in the rat brain 24 h after CPR. Considering the close relationship between mitochondria and ER, in the present study, we found that rats pretreated with Sal showed not only improved neurological performance with inhibition of neuronal apoptosis but also better cortical mitochondrial morphology 24 h after CPR. We further found that the beneficial effects could be attributed to retained mitochondrial function, as evidenced by decreased intracellular active species, an elevated MMP, and the inhibition of apoptotic pathways.

Salubrinal was first identified in 2005 as a selective inhibitor of ER stress [13]. At least some of the cell protective effects of Sal seem to be mediated by the induction of eIF2*α* phosphorylation without any effect on the transcription-dependent component of the UPR [13]. However, many studies have shown that the protective effect of Sal may be related to its ability to reduce apoptosis in brain tissue [10, 18, 20, 21]. In the current study, TUNEL staining and serum markers of neuronal damage (NSE and S100*β*) were used to detect brain damage after resuscitation. Consistent with our previous studies [10], Sal inhibited hippocampal neuronal apoptosis 24 h after CA. The Bcl-2 family of proteins plays a key role in intracellular apoptotic signal transduction. In cell apoptosis, the expression of antiapoptotic Bcl-2 is suppressed, and Bax is activated [22]. The activation of the caspase family of proteins and cell death along with the activation of Bax increased the release of cytochrome C from mitochondria. CCAAT-enhancer-binding protein homologous protein (CHOP) has been linked to ER stress-induced cell death and may act partly by inhibiting Bcl-2 [23, 24]. Consistent with these findings, in the present study, Sal pretreatment decreased Bax levels and increased Bcl-2 levels, resulting in the recovery of the Bcl-2/Bax ratio 24 h after CA. In addition, we found that Sal reduced the upregulation of the cleaved caspase 3 and caspase 12 proteins compared with the levels in the vehicle group [10]. Some previous studies have indicated that reduced CHOP levels may lead to increased Bcl-2 expression [23, 24]. On the other hand, Sal has been shown to exert its effects in nerve injury through increasing eIF2*α* phosphorylation and GRP-78 and ATF4 expression but strongly reducing CHOP and caspase 12 levels [20, 25]. This could be one of the mechanisms by which Sal reduces apoptosis.

Mitochondria are the major intracellular sources of reactive oxygen species (ROS). Oxidative stress is the main cause of systemic I/R injury. ROS-mediated oxidative damage to mitochondria favors more ROS production. According to reports, Sal can alleviate brain damage by reducing oxidative stress in a traumatic brain injury model [21]. Therefore, we next assayed SOD activity and MDA content in the cortex. Consistent with previous reports, we found that Sal restored SOD activity and reduced the MDA level in the cortex 24 h after CA. Therefore, our study showed that Sal pretreatment can reduce brain damage through the attenuation of oxidative stress after resuscitation.

Mitochondrial dysfunction plays an important role in brain damage after CPR. Many studies have demonstrated that the mitochondrial membrane potential (MMP) is a sensitive indicator that reflects mitochondrial function. In this study, we detected extreme hyperpolarization of the MMP that exceeded the normal potential 24 h after CA. Meanwhile, cytochrome C was elevated dramatically in the cytosol, which may have caused cell apoptosis. This indicated that an increased MMP after ROSC damages the integrity of the mitochondrial membrane. These results suggested that Sal reduces the release of cytochrome C and inhibits the hyperpolarization of the MMP after CA.

Hypoxia inducible factor (HIF) is a transcription complex that plays a central role during oxygen homeostasis. Among the HIF isoforms, HIF-1*α* is the most widely described in brain I/R injury [26, 27]. HIF-1*α* is a major transcription factor involved in the expression of genes involved in mitochondrial function, apoptosis, angiogenesis, glucose metabolism, and resistance to oxidative stress [28, 29]. Lopez-Hernandez et al. found that ER stress and the HIF-1 signaling pathways are involved in cortical neuronal damage caused by chemical hypoxia (CoCl_2_) [20]. In the present study, we found that Sal pretreatment increased the level of HIF-1*α* in the cortex. This finding is consistent with previous studies showing that enhanced HIF-1*α* expression reduces the neural cell apoptosis associated with cerebral I/R injury [20, 30].

We recognize several limitations of our study. First, this was a small animal study and was performed on healthy animals, while most CA patients have an underlying disease. Second, we only studied one time point (24 h). Further studies should extend the observation period to at least 72 h after ROSC. Third, we used a pretreatment model of Sal administration and tested only one dose of Sal, which is similar to previous reports [6, 13, 18]. However, further research is needed to study the optimal dose of Sal and the effects of posttreatment with Sal. Fourth, the ultrastructural analysis is presented without quantifications of mitochondrial characteristics that can lead to confusion in objective understanding of the differences between groups.

## Figures and Tables

**Table 1:** Physiological variables at baseline, 30 min after ROSC, and 10 min after MV weaning. Comparisons of the results of arterial blood gas analysis, HR, and MAP revealed no difference between the vehicle group and the Sal group by Student's *t-*test.

	Sham (*n* = 15)	Vehicle (*n* = 15)	Sal (*n* = 15)	*P* value
Before CA				
Body weight (g)	342.7 ± 15.8	345.9 ± 19.8	341.7 ± 12.2	0.76
pH	7.42 ± 0.03	7.42 ± 0.03	7.42 ± 0.02	0.99
PaCO_2_ (mmHg)	35.7 ± 1.8	35.9 ± 1.7	35.8 ± 1.5	0.95
PaO_2_ (mmHg)	99.3 ± 0.7	99.4 ± 0.7	99.7 ± 0.6	0.39
Lactate (mmol L^−1^)	0.6 ± 0.2	0.6 ± 0.1	0.6 ± 0.2	0.92
Base excess	−3.5 ± 0.4	−3.5 ± 0.3	−3.7 ± 0.4	0.28
Mean arterial pressure (mmHg)	96.9 ± 5.4	96.5 ± 4.5	94.7 ± 5.4	0.47
Heart rate (bpm)	365.0 ± 22.1	350.6 ± 27.2	362.9 ± 24.1	0.24
Body temperature	37.0 ± 0.16	36.9 ± 0.10	37.0 ± 0.12	0.32
30 min after ROSC				
pH	N/A	7.33 ± 0.04	7.32 ± 0.05	0.74
PaCO_2_ (mmHg)	N/A	40.9 ± 5.1	41.7 ± 5.1	0.67
PaO_2_ (mmHg)	N/A	410.0 ± 44.9	399.7 ± 50.0	0.56
Lactate (mmol L^−1^)	N/A	6.2 ± 1.2	6.4 ± 1.3	0.53
Base excess	N/A	−9.9 ± 3.7	−9.8 ± 3.2	0.92
Mean arterial pressure (mmHg)	N/A	79.8 ± 6.6	79.6 ± 5.2	0.93
Heart rate (bpm)	N/A	305.7 ± 35.2	308.4 ± 36.1	0.84
Body temperature	N/A	37.0 ± 0.12	37.0 ± 0.10	0.74
10 min after weaning of MV				
pH	N/A	7.35 ± 0.04	7.34 ± 0.04	0.80
PaCO_2_ (mmHg)	N/A	40.3 ± 4.4	40.5 ± 5.7	0.91
PaO_2_ (mmHg)	N/A	387.3 ± 52.0	370.8 ± 43.8	0.35
Lactate (mmol L^−1^)	N/A	2.7 ± 0.7	2.5 ± 0.7	0.34
Base excess	N/A	−5.9 ± 1.5	−5.9 ± 1.8	0.97
Mean arterial pressure (mmHg)	N/A	92.3 ± 10.3	91.1 ± 8.9	0.75
Heart rate (bpm)	N/A	331.1 ± 37.3	340.9 ± 37.6	0.47
Body temperature	N/A	36.9 ± 0.19	36.9 ± 0.20	0.55

## Data Availability

The data used to support the findings of this study are included within the article.
